# PATHWEIGH, pragmatic weight management in adult patients in primary care in Colorado, USA: study protocol for a stepped wedge cluster randomized trial

**DOI:** 10.1186/s13063-021-05954-7

**Published:** 2022-01-10

**Authors:** Krithika Suresh, Jodi Summers Holtrop, L. Miriam Dickinson, Emileigh Willems, Peter C. Smith, R. Mark Gritz, Leigh Perreault

**Affiliations:** 1 Colorado School of Public Health, 13001 East 17th Place, Aurora, CO 80045 USA; 2 University of Colorado School of Medicine, 13199 E Montview Blvd Ste 300, Aurora, CO 80045 USA

**Keywords:** Cluster randomized trial, Mixed methods, Pragmatic trial, PRISM, RE-AIM, Stepped wedge, Weight loss

## Abstract

**Background:**

Despite the overwhelming prevalence and health implications of obesity, it is rarely adequately addressed in a health care setting. PATHWEIGH is a pragmatic approach to weight management that uses tools built into the electronic medical record to overcome barriers and guide care. Implementation strategies are employed to facilitate adoption and use of the PATHWEIGH tools and processes. The current study will compare the effectiveness of PATHWEIGH versus standard of care (SOC) on patient weight loss in primary care and explore factors for its successful implementation.

**Methods:**

A stepped wedge cluster randomized trial design will be used within an effectiveness-implementation hybrid study. Adult patient weight loss and weight loss maintenance will be compared in PATHWEIGH versus SOC in 57 family and internal medicine clinics in a large health system in Colorado, USA. Effectiveness will be evaluated using generalized linear mixed models to determine statistical differences in weight loss and weight loss maintenance at 6, 12, and 18 months. Patient-, provider-, and clinic-level predictors will be identified using mediator and moderator analyses. Conceptually guided by the Practical, Robust, Implementation and Sustainability Model (PRISM), a mixed methods approach including quantitative (practice surveys, use tracking) and qualitative (interviews, observations) data collection will be used to determine factors impeding and facilitating adoption, implementation, and maintenance of PATHWEIGH and evaluate specified implementation strategies. A cost analysis of the practice and system costs and resources required by PATHWEIGH relative to the reimbursement collected will be performed.

**Discussion:**

The effectiveness and implementation of PATHWEIGH, and their interrelatedness, for patient weight loss are collectively the focus of the current trial. Findings from this study are expected to serve as a blueprint for available and effective weight management in primary care medical practice.

**Trial registration:**

ClinicalTrials.govNCT04678752. Registered on December 21, 2020.

**Supplementary Information:**

The online version contains supplementary material available at 10.1186/s13063-021-05954-7.

## Background

In the USA, obesity prevalence continues to increase. In 2019, the Centers for Disease Control and Prevention (CDC) estimated that 70% of US adults were considered overweight and 42% had obesity [1]. Obesity is a well-established risk factor for numerous diseases, including diabetes, cardiovascular disease, cancer, immobility, mental health issues, as well as all-cause mortality [2]. Obesity is also a strong and independent risk factor for poor outcomes due to COVID-19 [3]. In 2018, direct and indirect costs associated with obesity in the USA reached $1.7 T [4]. Importantly, obesity is being increasingly recognized not only as a risk factor for disease, but a disease unto itself [2]. Despite this, obesity is rarely addressed in a healthcare setting. Only ~ 50% of people with a BMI of 50 kg/m^2^ (considered morbid or extreme obesity) have a documented diagnosis of obesity [5] and < 1% of people with any degree of overweight or obesity are offered anything other than lifestyle advice [6].

Reasons behind the lack of weight management prioritization in clinical settings are extensive and complex. Health care providers cite lack of time, education, and resources, as well as competing issues, as the leading reasons why obesity is not prioritized [7–9], but poor reimbursement and lack of effective tools are also widely cited [7, 8]. Further, lack of data on long-term effectiveness of weight loss treatments leads to uncertainty and learned helplessness among both health care providers and patients. Eighty-two percent of people with obesity believe they are responsible for their weight; a number highly corroborated by health care providers, enhancing shame and hesitancy to seek help [7]. While these are only a few of the reasons why weight is rarely addressed, increasing evidence suggests barriers can be overcome by intentionally employing long-term evidence-based strategies. Importantly, such strategies must be employed where patients access care, most notably in primary care.

Primary care is undergoing rapid transformation with a shift toward population-based approaches that prioritize preventive care. Initiatives such as pay for performance, the Patient-Centered Medical Home [10], and efforts associated with the Affordable Care Act [11] have brought a host of structures, tools, and processes not previously available, creating new opportunities to engage patients in efficacious programs. Many practices now have electronic medical records (EMRs), patient registries, and a team approach to care that may include expanded care visits with care managers, dietitians, and social workers or psychologists [12, 13]. Increasingly, value-based payment models incentivize practices to identify patients at high risk for complications of chronic conditions and to mitigate these risks. Many of these chronic conditions can be improved or alleviated by reducing excess weight.

Importantly, patient demand is high for weight management in primary care*.* A study by Sherson et al. [14] reported that the majority of patients want to discuss weight loss with their physicians. Specifically, patients value physician direction with their diet, physical activity, and goal setting [15]. In addition, recent years have ushered in numerous and diverse options for weight management that extend significantly beyond lifestyle advice. Medications for weight loss are being more extensively studied and demonstrating improved outcomes [16–19]. Bariatric surgery provides a possible option to address weight and reverse potentially life-threatening conditions such as heart disease and diabetes in both adolescents and adults [20–23]. Intensive behavioral therapy (IBT) for obesity is now a covered benefit under Medicare [24]. Together, primary care practice transformation, reimbursement for obesity care, and better therapies for weight management suggest new and pragmatic approaches can emerge.

In the Fall of 2020, the National Institutes of Health (NIH) National Institute of Diabetes and Digestive and Kidney Diseases (NIDDK) funded “PATHWEIGH: pragmatic weight management in primary care” (1R18DK127003). The aim of PATHWEIGH is to create the blueprint for pragmatic, scalable, and sustainable weight management in primary care using existing resources. To achieve this aim, this study will deploy an effectiveness-implementation hybrid type 1 [25] stepped wedge cluster randomized trial design [26] in 57 health-system owned family medicine and general internal medicine clinics that span diverse settings, patient populations, and community contexts in Colorado.

## Aims and hypotheses

The specific aims of the current research are to:

1. Compare the effectiveness of PATHWEIGH versus standard of care (SOC) on weight loss and weight loss maintenance.
2. Identify patient, provider, and clinic-level predictors that are associated with weight loss and weight loss maintenance.
3. Describe factors associated with practice adoption, implementation, and maintenance of PATHWEIGH.

We hypothesize that PATHWEIGH will lead to greater weight loss at 6 months (primary) and greater weight loss maintenance at 12 and 18 months (secondary) compared to SOC [Hypothesis I]. Key patient, clinician, and clinic characteristics will mediate and moderate patient weight loss and weight loss maintenance [Hypothesis II]. Effective implementation will be vital to anticipated implementation and clinical outcomes [Hypothesis III].

## Methods

The study methods are described in compliance with the SPIRIT statement [27] and the Consolidated Standards of Reporting Trials (CONSORT) extension for stepped wedge cluster randomized trials [28] to ensure appropriate reporting of the study protocol and completed trial. The SPIRIT checklist is provided in the Additional file.

### Study setting

**Fig. 1:**
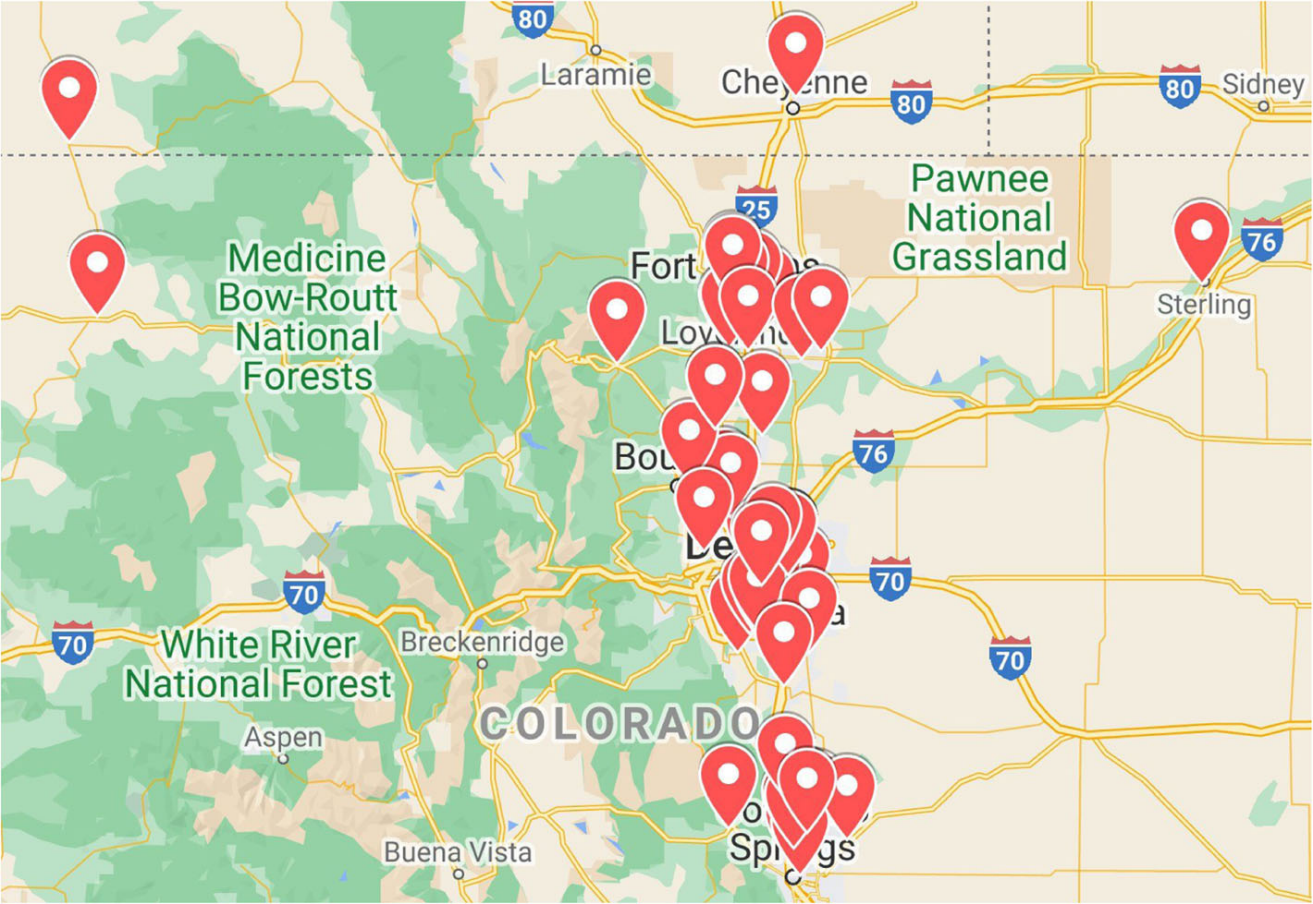
Location of participating Colorado and Wyoming clinics. Image created using Google Map data 2022

This study will be conducted in 57 family medicine and general internal medicine clinics within a large, multi-specialty health care system in Colorado (Fig. 1). One primary care clinic was excluded as it was a pilot test site [29]. These practices are comprised of approximately 400 clinician full-time equivalents (physician, nurse practitioner, physician assistant) caring for nearly 500,000 patients in diverse geographic and socioeconomic contexts. All practices have the following resources for the implementation of obesity management: access and full use of the Epic EMR, practice quality improvement facilitators, standardized workflows for medical assistants, funds for site innovation, inter-connectedness to the system at large, and regular training opportunities. Primary care leadership has unanimously offered their support for this project.

### Design and randomization

**Fig. 2:**
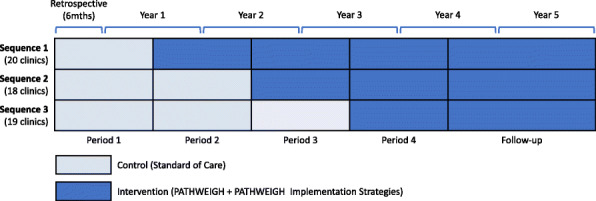
Study diagram for the PATHWEIGH study, a stepped-wedge cluster hybrid type 1 randomized controlled trial design

We will use a type 1 hybrid effectiveness-implementation stepped wedge cluster randomized design to assess both patient-level clinical and practice-level implementation outcomes [25, 26]. The intervention will be implemented sequentially in the 57 participating clinics in three steps over a 4-year period (Fig. 2). All sites will start in the control condition of SOC for weight management. Every year, one sequence of clinics will transition to the intervention condition after which the use of PATHWEIGH will be encouraged for all weight-prioritized visits and corresponding PATHWEIGH implementation strategies will be provided. Clinics were randomly assigned by the study statistician to each sequence using covariate constrained randomization [30] to achieve balance on clinic type (academic, affiliate, non-academic), clinic location (urban/suburban vs. rural), provider type (family medicine (FM), general internal medicine (GIM), or both), annual number of patient visits, and patient insurance status (percentage Medicaid) (Table 1). A stepped wedge extension for this procedure was used to account for the proportion of the study period that each of the sites spend in the control and intervention periods [30]. For additional details about the randomization procedure see Additional file.

**Table 1:** Characteristics of participating practices and patients by randomization sequence

Site characteristic	Sequence 1 (20 clinics)	Sequence 2 (18 clinics)	Sequence 3 (19 clinics)
**Type,** *n*			
Academic	1	1	2
Affiliate	4	2	2
Non-academic	15	15	15
**Location,** *n*			
Urban	17	16	16
Rural	3	2	3
**Specialty,** *n*			
Family Medicine only (FM)	10	9	11
General Internal Medicine only (GIM)	6	6	4
Mixed FM/GIM	4	3	4
**Number of patients visits in 2019,** median (IQR)	10,589 (6059–22,233)	13,579 (7666–20,035)	9815 (3552–29,910)
**Percentage Medicaid,** median (IQR)	3.5 (2.0–5.0)	5.0 (3.0–7.0)	2.5 (1.8–7.0)

### Participant eligibility criteria

Patients eligible for this analysis must (1) be aged 18 years or older, (2) be overweight (BMI ≥25 kg/m^2^) at initial visit, and (3) have had at least one weight-prioritized visit in a participating clinic. Exclusion criteria in the original protocol include limited cognitive ability, have a non-home residence, or have less than 1-year life expectancy. Ultimately, these were not applied due to the inability to obtain the necessary fields in the data extraction; however, the number of individuals that would be excluded based on these criteria is expected to be minimal and balanced across the study arms. Weight-prioritized visits are defined as the chief complaint or reason for the visit being at least one of the following: (1) “overweight,” “obesity,” or “weight,” (2) ICD-10-CM codes for billing E66–E.66.9, Z68.25–45, (3) use of the “obesity brief HPI” Epic flowsheet, or (4) the PATHWEIGH patient questionnaire.

### Data collection and management

**Fig. 3:**
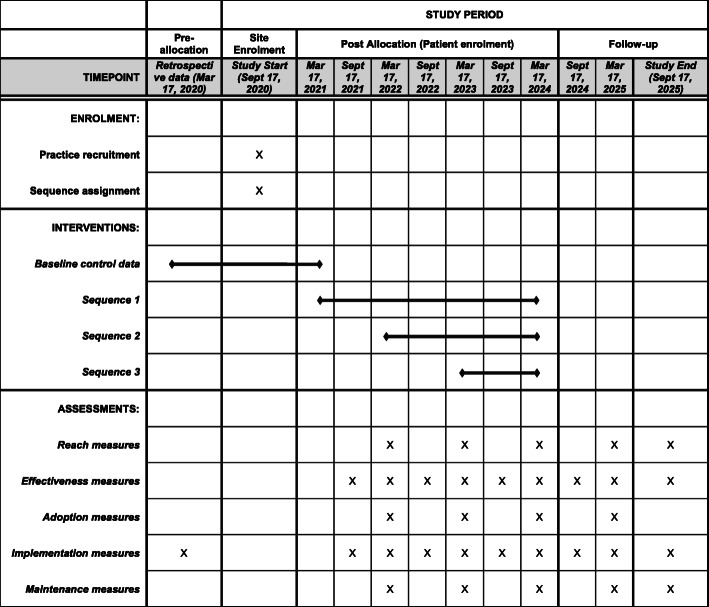
SPIRIT diagram

De-identified data will be extracted from Epic (the electronic medical record) from March 17, 2020, to March 16, 2021, then every 6 months from March 17, 2021, until September 17, 2025. The study timeline is depicted in a SPIRIT diagram in Fig. 3. Data extraction will be performed by Health Data Compass, the Colorado Center for Personalized Medicine’s cloud-based enterprise data warehouse and management platform. Health Data Compass extracts de-identified data using a proprietary process, wherein all data are checked for process compliance and missingness prior to release to the study team. As these data are de-identified, informed consent was not utilized. However, a written informed consent process was utilized for specified, individually contacted patients and practice members for the purposes of the implementation evaluation.

Retrospective data will be collected on all patient encounters at the 57 primary care clinics at the specified times described above. Specifically, how many patient visits occurred at each clinic, how many discrete patients were seen at each clinic, how many patients were ≥18 years old with a BMI ≥ 25 kg/m^2^, and how many patients who were ≥18 years old with a BMI ≥25 kg/m^2^ had a weight-prioritized visit (either in person or by telehealth). These data will be illustrated in an Expanded CONSORT diagram [31]. Data will be stored on a secure data server with access provided to the study statisticians. The statisticians will not be blinded to the assignment of the interventions.

### Sample size

A conservative sample size estimate is 6840 total patients (120 patients per clinic from 57 clinics) that assume a minimum of 30 eligible patients per clinic per year based on the smallest clinic. We present the power calculations assuming an intraclass correlation coefficient (ICC) between 0.02 and 0.05. With this design, we will have 80% power (alpha=0.05, two-sided) to detect a standardized effect size of 0.118 (ICC=0.02) to 0.121 (ICC=0.05). For our primary effectiveness outcomes, we estimate a standard deviation (SD) of 10.1 percentage points for percentage change in weight using our pilot study, and a similar SD estimate of 10.7 for absolute (kg) change in weight. Thus, with 80% power we can detect a mean difference in weight change of 1.19 (ICC=0.02) to 1.22 (ICC=0.05) percentage points between the control and intervention conditions, and a mean difference in absolute weight change of 1.26 kg (ICC=0.02) to 1.29 kg (ICC=0.05).

### Interventions

#### Current standard of care (SOC)

SOC will be the control condition that patients will receive during the control phases of the design. Clinics in the SOC condition will conduct visits in which weight may be discussed (defined as “weight-prioritized” for purposes of data extraction) without access to either the PATHWEIGH tools in Epic or the PATHWEIGH implementation strategies. Current SOC for obesity in the primary care clinics is called “obesity – brief history of present illness (HPI),” and the option to select “Obesity brief HPI” is currently available to all Epic users at all practices. For an example of the Obesity brief HPI questionnaires, see Additional File Fig. S1. For a weight prioritized visit, this entails a brief historical intake of a patient’s weight and is intended to initiate a discussion regarding potential strategies for weight loss. Because SOC can be highly variable, we will conduct fidelity checks by performing sensitivity analyses for the use of the “Obesity brief HPI” in the SOC clinics at our every 6-month data extractions. A change in the use of the “Obesity brief HPI” by more than 30% between 6-month intervals will be investigated. There are currently no systematic weight management interventions at the clinics.

#### PATHWEIGH: Clinical workflow and management

**Fig. 4:**
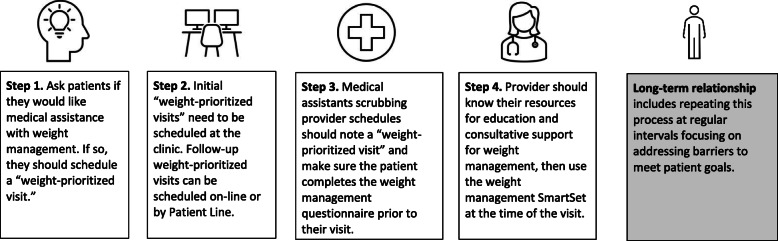
PATHWEIGH clinic flow

PATHWEIGH is a workflow optimization and management system for weight management built into Epic (Fig. 4). The PATHWEIGH tools in Epic are designed to address numerous barriers to prioritizing weight management in primary care [8]. Once practices transition to receive the intervention, PATHWEIGH will be “turned on” within Epic and the PATHWEIGH implementation strategies will be provided. The key components of PATHWEIGH are summarized in Table 2 and include (1) encouraging patients to schedule a weight-prioritized visit if they would like medical assistance with weight management, (2) improving workflow efficiency around weight-prioritized visits, and (3) clinical decision support during weight-prioritized visits.

**Table 2:** Key components of PATHWEIGH and PATHWEIGH implementation strategies

***PATHWEIGH is a weight management care path built into Epic.*** It includes: • Weight-prioritized visit type: Defines the scope of the visit for the patient and provider. • Time-efficient documentation and data capture: Patient questionnaire completed prior to the visit captures history around weight gain and barriers to weight loss that can guide the patient-clinician conversation and treatment plan. • Clinical decision support: SmartSet in Epic suggests weight loss approach(es) based on patient BMI and preferences according to the Obesity Society guidelines [32]. • Tracking: Displays progress with weight loss and weight loss maintenance during patient visits to guide the patient-clinician conversation and evolving treatment plan over time. • Diagnosis and billing: Tool in Epic automatically adds the diagnosis of overweight (for BMI 25-29.9 kg/m^2^) or obesity (for BMI> 30 kg/m^2^) and links the most common weight-related co-morbidities (according to the Center for Medicare and Medicaid Services) to ensure adequate provider reimbursement.	
***PATHWEIGH implementation strategies*** include: • Practice facilitation: Practice facilitators drive implementation efforts by working with practice-selected Practice Champions, engaging leadership in goal setting and monitoring along system and practice goals, and utilizing quality improvement techniques for supporting adoption, implementation, and management of PATHWEIGH. • Clinician education: Online e-learning module on evidence-based weight management treatment which includes 2 credits of continuing medical education. • Consultation support: built-in to Epic consultation support for clinicians by request; built-in to Epic availability of UpToDate, a subscription-based clinical reference.	

#### Staff workflow

Placards may be placed at the front desk of the clinics alerting patients that they may make a weight-prioritized visit if they would like medical assistance with their weight. Each clinic or provider will determine the number of weight-prioritized visits available. Once a patient schedules a weight-prioritized visit, Epic will request they complete a specified weight management questionnaire upon e-check-in prior to their visit. Medical assistants (MAs) will review provider schedules ahead of time to ensure patients complete this questionnaire prior to their visit. At the time of the patient visit, MAs will record the patient’s weight and vital signs with no other assistance required. Collectively, the workflow maximizes time for the clinician-patient conversation in the context of the Patient-Centered Medical Home [10].

#### Clinician workflow

To optimize efficiency and guide care, health care providers are encouraged to use the PATHWEIGH weight management SmartSet in Epic. A SmartSet is a documentation template used in the ambulatory setting comprised of a group of orders and other elements, such as notes, chief complaints, medications, and levels of service charges, that are commonly used together to document a specific type of visit. This SmartSet addresses the following.

1. Diagnosing overweight or obese patients with a BMI > 25 kg/m^2^ or > 30 kg/m^2^, respectively, recognizing these as bona fide disease states [6]
2. Linking diagnoses for weight-related comorbidities to facilitate reimbursement
3. Pre-populating information from the patient weight management questionnaire to serve as documentation and to guide the clinician-patient discussion, and flagging weight management strategies based on guidelines from the Obesity Society [32], specifically, the discussion of lifestyle change for patients with BMI ≥ 25 kg/m^2^, consideration of medications impacting obesity as well as anti-obesity medication at BMI ≥ 30 kg/m^2^ (or BMI ≥ 27 kg/m^2^ with weight-related comorbidities) and consideration of bariatric surgery for BMI ≥ 40 kg/m^2^ (or BMI ≥35 kg/m^2^ with weight-related comorbidities)
4. Providing options for orders that include laboratory testing, referrals, or procedures

Behavioral health professionals work alongside many of the primary care providers and are encouraged adjuncts to facilitate weight loss. Use of the PATHWEIGH tools is entirely optional for clinicians. Use may be deployed by the clinicians in advance of the visit or if they or the patient spontaneously decides to prioritize weight management. The process strongly encourages regular follow-up (usually at 4–6 week intervals and more often initially). A key advantage is that the clinician is the patient’s regular provider and will have an ongoing relationship with the patient over time and can safely manage or discontinue medications or other issues for weight-related comorbidities.

### Implementation framework and strategies

#### Conceptual framework

Implementation and evaluation of PATHWEIGH is guided by the Practical, Robust, Implementation and Sustainability Model (PRISM) [33]. RE-AIM (Reach, Effectiveness, Adoption, Implementation, and Maintenance) has often been used as a planning and evaluation framework for capturing important measures in implementation research, but it lacks measures of context and drivers of success [34–37]. PRISM was developed to address the issues of multi-level features for successful program design, predictors of implementation success, factors associated with diffusion, implementation, and maintenance [33]. The key contextual factors for examination include the interaction of system and patient levels with the intervention (includes model components of recipients and intervention), infrastructure elements related to implementation and sustainability (model element of implementation sustainability and infrastructure), and external/environmental factors (model element of external environment) and how they exert an influence on the RE-AIM outcomes.

### Implementation strategies

Implementation strategies are interventions designed to improve the effectiveness of PATHWEIGH by promoting its adoption and use [38, 39]. These strategies were based on our development work, where key stakeholders identified the following three main areas of focus for PATHWEIGH implementation: (1) training using an e-learning module including continuing medical education (CME) credit for providers on how to counsel patients on weight loss and how to prescribe evidence-based weight loss treatments, (2) consultation support by experts in weight management built into Epic, and (3) assistance from practice facilitators available to each clinic to identify clinic champions and to assure that PATHWEIGH gets incorporated into workflows, becomes known to patients, and is monitored using quality improvement (QI) principles. All implementation strategies are deployed to clinics when they are randomly assigned to begin using PATHWEIGH. These are common resources available in other health systems to facilitate eventual dissemination.

## Evaluation

### Aim 1: Compare the effectiveness of PATHWEIGH versus standard of care (SOC) on weight loss and weight loss maintenance.

#### Outcomes and Measures

The primary outcome is patient weight between baseline and 6 months in kilograms (kg) as measured by a standard scale. The secondary outcome of weight loss maintenance will be defined at 12 and 18 months as a binary outcome of weight increase ≤ 10% from the patient’s 6-month weight. Weight outcomes will be extracted from the EMR. The primary independent variable will be the intervention status, which will be determined based on visit date and practice sequence. Additional covariate data will be collected from the EMR on potential confounders at the level of patient, provider, and practice. Patient-level data will include demographic information, health metrics, behaviors and goals, and treatment strategy employed. Provider-level data will include demographic information, years practicing, and percent time practicing. Clinic-level data will include site type/discipline, location, and size. Outcomes, measures, and timing of data collection are described in Table 3.

**Table 3:** Summary of outcomes, measures, data sources, and timing of collection for PATHWEIGH study

Construct	Metric/measure	Method of collection	Timing
**Patient-specific outcomes**			
Weight-related outcomes	Weight (kg), height (cm), weight loss maintenance (weight increase ≤ 10%)	EMR	Collected during routine care
Clinical and laboratory values	Thyroid-stimulating hormone (TSH), lipid panel, liver function tests, A1c, blood pressure	EMR	Collected during routine care
Patient characteristics	Demographics (age, gender, race/ethnicity, insurance, comorbidities, medications)	EMR	Collected during routine care
**Clinician and clinic staff-specific outcomes**			
Clinic characteristics	# and characteristics of clinicians and patients empaneled to the clinic	Survey to practice leadership	Baseline
Practice culture	Practice culture scale [40, 41]	Survey to providers and staff	Baseline and 1-year post-intervention
Implementation climate	Implementation climate scale [42, 43]	Survey to providers and staff	Baseline and 1-year post-intervention
History of practice improvement	# and type of practice improvement in the past year	Survey to providers and staff	Baseline and 1-year post-intervention
Self-efficacy and satisfaction	One item assessment of perceived confidence in ability to provide weight management, one item assessment of perceived satisfaction	Survey to providers and staff	Baseline and 1-year post-intervention
**RE-AIM Outcomes**			
**Reach** – The absolute number, proportion, and representativeness of individuals who participate	#/characteristics of patients in PATHWEIGH out of weight-prioritized and eligible per clinic	EMR	Baseline (patient characteristics) and at each 1-year step (patient participation)
	Barriers, facilitators to participation and outcomes	Interviews with selected patients	6-month post-intervention start for the patient
**Effectiveness** – The impact of an intervention on important patient outcomes	Evaluated using weight-related outcomes described above.		
**Adoption** – The absolute number, proportion, and representativeness of settings and providers who initiate the intervention	Use of PATHWEIGH by any provider in a clinic (setting level) and each provider (provider level)	Survey to practice leadership (practice characteristics), EMR (provider use of PATHWEIGH)	Baseline and at each 1-year step
	Factors influencing adoption/implementation	Interviews with selected providers and staff	Baseline and 1- year post-intervention start for each sequence
**Implementation** – The intervention agents’ fidelity to the various elements of the intervention’s protocol including consistency of delivery as intended and the time and cost of the intervention	Fidelity to PATHWEIGH functional core components and SOC in control clinics; use of implementation strategies; Clinician use of PATHWEIGH for > 50% weight-prioritized visits; e-learning module completion per clinician; use of consultation; participation in PF trainings	EMR	Ongoing collection; review at 6-month post-intervention start and study end; completion of implementation strategies
	Acceptability of PATHWEIGH; adaptations	Survey to all providers, some staff, and selected patients	6- month post-intervention start for that cohort
	Barriers, facilitators to implementation	Interviews with selected providers, clinic leaders, and staff	1-year post-intervention start for each cohort
	Cost and resources for implementation	Cost/resources interviews with practice managers and selected provider/staff by research assistant	Control condition and 1-year post-intervention start for each cohort
**Maintenance** - The extent to which the intervention becomes part of practice	Plans to continue/adapt PATHWEIGH past study	Survey and interviews with practice managers and selected providers and staff	1-year post-intervention start for each cohort; and study end

Abbreviations: *EMR* electronic medical record, *PF* practice facilitator, *SOC* standard of care

#### Analysis

A generalized linear mixed modeling strategy will be employed to account for multiple levels of correlation (i.e., between patients within practices and between measurements on a single patient over time) [44]. This approach allows for the most efficient use of data if any follow-up measurements are missing for some subjects. We will follow an intention-to-treat approach for our primary analysis, i.e., patients will be analyzed according to their clinic’s intervention condition (PATHWEIGH vs. SOC) at the time of the patient’s index visit. An index visit will be defined as the patient’s first weight-prioritized visit in an intervention condition. Follow-up visits at which weight is recorded will be associated with the intervention status of the patient’s preceding index visit.

For the primary analysis, although the parameter of interest is weight change at 6 months, weight collected from baseline to approximately 9 months post-baseline for the individual patient will be included in the primary analysis because patients may not have visits with recorded weight within the 6-month time frame. The mixed model will include random intercepts for patient, provider, and clinic, along with fixed effects for time and intervention condition. The time effects are two-fold: (i) calendar time, divided into four 1-year periods and included as a categorical variable to flexibly model its effect on the outcome and account for temporal trends, and (ii) individual patient time, included as a continuous variable (i.e., time from baseline) to accommodate varying visit times determined by each patient’s visit schedule. The intervention condition variable will be a binary indicator that is equal to zero if the patient’s index visit occurs at a clinic when it is in the control condition and becomes equal to 1 when the patient has an index visit at a clinic after it has crossed into the intervention condition. Patient-, provider-, and practice-level covariates will be included in models if deemed to be potential confounders in univariate models. An additional fixed effect for the interaction between patient time and intervention condition will be used to estimate the parameters of primary interest, i.e., the difference in change in outcomes over time between the intervention and control conditions. The primary analysis will test the null hypothesis that the interaction effect coefficient is equal to zero to determine whether there is a significant difference in weight change between the PATHWEIGH and SOC conditions. Using the model, we will report the mean weight change in the control and intervention groups and their difference at 6 months, with corresponding 95% confidence intervals and *p* values.

To examine the secondary binary outcomes of weight maintenance (defined as < 10% weight gain at 12 and 18 months), we will use generalized linear mixed models. Since this analysis will involve a single outcome per patient, the model will only include a random intercept for provider and clinic. The test of intervention effectiveness will be captured by the fixed effect term for the intervention condition.

The primary analysis is expected to provide a conservative estimate of the treatment effect, and we propose the following secondary analyses to assess the treatment effect in alternative cohorts. We will perform a per-protocol analysis that uses the described models to compare those that received PATHWEIGH to those with a SOC weight-prioritized visit. This analysis will provide a better estimate of the efficacy of the intervention by including only those in the PATHWEIGH condition that were considered to satisfy the criteria for receiving the intervention as assessed by the provider’s use of at least one of a weight prioritized visit “type”, the patient weight management questionnaire, and/or the weight management SmartSet. Additionally, we will perform an analysis using all those that are eligible for PATHWEIGH based on the criteria of age and BMI, regardless of whether they had a weight-prioritized visit. This analysis will allow us to account for the potential selection bias induced by the implementation of PATHWEIGH potentially increasing the number of weight-prioritized visits and thus increasing the enrollment of patients in the intervention period that differ systematically from those in the control period.

### Aim 2: Identify patient, provider, and clinic-level predictors that are associated with weight loss and weight loss maintenance

#### Outcomes and measures

Patient-level predictors will be captured from the EMR for each visit. Potential patient-level moderators will include age, sex, race/ethnicity, insurance status, baseline BMI, baseline use of medication that may contribute to weight gain, and score > 9 on Patient Health Questionnaire-8 or -9 (PHQ8, PHQ9), or Generalized Anxiety Disorder-7 (GAD7). Patient-level mediators will include treatment prescribed, in particular prescription of anti-obesity medication and performance of an endoscopic or surgical procedure with bariatrics. Provider-level predictors that can be obtained from EMR include provider age, sex, years in practice, and percent time performing clinical duties. Practice-level predictors include patient and provider number and demographics for each, as well as payor mix.

#### Analysis

Moderation will be evaluated by including an interaction term between the treatment variable and possible moderating variables in the outcome mixed effects regression models used in Aim 1. A significant interaction effect will conclude that the considered variable moderates the effect of PATHWEIGH on the outcome. Mediation analysis will be conducted using the multi-level modeling framework to account for clustering within clinics and providers [45, 46]. Clinic- and provider-level mediators will be restricted to those that can be collected from EMR in both the control and intervention phases. Specifically, we will fit two models: (1) a mediator model to describe the effect of the treatment on the mediator, and (2) an outcomes model to describe the effect of the mediator on the outcome, adjusting for the treatment. Additionally, to be considered a mediator, change in the mediator variable must occur prior to or during the period of patient-level change. Both models will be adjusted for relevant patient-, provider-, and clinic-level variables. The outcomes model will additionally adjust for a time effect to account for temporal effects in the stepped wedge design. These models will be adjusted to include the appropriate random effects and variables based on the level of the considered mediator variable (i.e., patient, provider, clinic). The mediation effect will be summarized as the product of the treatment coefficient from the mediator model and the mediator coefficient from the outcomes model. We will test the null hypothesis that this product is equal to zero, and a statistically significant result will indicate that there is strong evidence that the effect of the treatment on the outcome is mediated by the considered variable. If necessary, we will explore more complex multi-level structural equation models [47].

### Aim 3: Describe factors associated with practice adoption, implementation, and maintenance of PATHWEIGH.

#### Outcomes and Measures

##### Practice member surveys

A baseline (pre-intervention) survey will be given to all practice members to assess personal characteristics (PRISM element of recipients) and practice culture, implementation climate, and perception of system priority for weight management (PRISM elements of implementation and sustainability infrastructure, and intervention). Clinicians will also be assessed on current weight management practices and their self-efficacy and satisfaction with obesity treatment (PRISM elements of recipients and intervention). These surveys will be repeated at 1 year after PATHWEIGH implementation for comparison pre-post intervention.

##### Provider, staff, and patient interviews

A purposeful selection of providers and staff, estimated at three to six total per practice, will be interviewed to assess RE-AIM outcomes. Practice staff and clinician interviews will focus on factors influencing adoption of PATHWEIGH and use of the PATHWEIGH implementation strategies, as well as influences on appropriate use of PATHWEIGH. Interviews will seek to understand the context of the delivery as well as the mindsets and belief systems driving thoughts and actions about what happens. External influences such as financial demands and staff turnover will be explored as potential sources of implementation difficulty (PRISM elements of recipients, intervention, implementation and sustainability infrastructure, external environment). Patient interviews (estimated at 40 total per year for each of the three cohorts) will explore their perspectives and experience with the intervention.

##### Other data collection

Observations will be conducted by research assistants at 3-6 months post-implementation. Research assistants will shadow selected patient visits to determine fidelity and quality of patient-provider communication on weight. A fidelity checklist and extensive field notes will be used. General practice observations will also be conducted to illuminate how the implementation works in practice and where there may be leakage, slippage, or gaps occurring in care processes in the intervention. Use of PATHWEIGH will also be extracted from Epic during the scheduled data query periods to determine clinicians’ use of PATHWEIGH tools as a measure of adoption. Additionally, research assistants will conduct key informant interviews with selected staff in practices to assess the cost and resources needed to implement PATHWEIGH and the associated implementation strategies. To assess financial sustainability, clinical billing data will be extracted from Health Data Compass to assess reimbursement for the weight-prioritized visits using standardized Medicare reimbursement rates.

#### Analysis

Quantitative analysis of the reach, adoption, implementation, and maintenance outcomes in Table 3 will be conducted using survey responses and fidelity checklists. Data will be analyzed descriptively (e.g., means, proportions) and estimated pre- and post-intervention. Inferential comparisons regarding association of the intervention with outcomes will be based on paired t-tests (continuous variables), McNemar’s chi-square tests (categorical variables), or generalized mixed modeling to adjust for potential confounders.

Qualitative methods, including interviews and observations, will be used to determine contributions to the adoption, implementation, and maintenance outcomes and PRISM contextual factors affecting these outcomes. Interviews will be audio-recorded, transcribed, cleaned, and entered into the qualitative software program, ATLAS.ti. We will use a grounded theory hermeneutic editing approach [48] to identify issues regarding intervention implementation from the perspectives of providers, staff members, leaders, and patients. Codes will be identified and created based on emergent elements in the data and will be used to tag relevant text from the transcripts. Quotation reports, which list all the associated quotations verbatim, will be generated and then organized by practice. The qualitative core group will hold ongoing meetings to read through all the quotations for these codes and categorize text that exemplifies the identified constructs. After initial thematic groupings, member-checking will be completed with key practice members. Additionally, we will use a matrix approach to determine patterns of emergent concepts across PRISM contextual factors and across practices.

Using a mixed methods concurrent parallel design, each element of data collection will occur separately [49]. Specifically, quantitative data will be collected and analyzed, and the qualitative data will be collected and analyzed. Then the two sets of data will be analyzed and interpreted together. Specific methods will include both a matrix approach and a data transformation approach to mixing of the data. Joint display tables describing the interplay of the quantitative and qualitative results will be created [50]. To achieve our aims, we will use the analysis technique of qualitative comparative analysis (QCA) [51]. QCA applies set theory to identify *necessary* and one or more *sufficient* sets of conditions to produce favorable outcomes, in this case, to determine what sorts of practice conditions and structures were needed to effectively implement PATHWEIGH [51, 52]. The fuzzy-set variant (fsQCA) allows simultaneous use of qualitative and quantitative data. It is newly applied to practice transformation [53], and we are using it in other work [54]. We will use this analysis to merge information from all sources and evaluate the contribution of specific PRISM factors on the outcome of intervention implementation and our practice break-even measure from the cost analysis. We will use the process detailed by Rihoux and Ragin and the fsQCA software [51].

### Return on investment

A formal return on investment (ROI) analysis will estimate the cost associated with adopting, implementing, and maintaining the intervention in practices and compare this to the difference in reimbursements before and after implementation in each practice. We will adapt a time-driven activity-based costing questionnaire that we have used in several previous studies [55, 56] for the estimation of the cost of staff time used in delivering the SOC and PATHWEIGH visits (including time of PATHWEIGH consulting obesity certified providers), practice facilitation support, and time clinic staff participate in training. In addition, we will measure the cost of materials provided to patients, hiring costs (if any), and other administrative costs incurred for all aspects of PATHWEIGH. To generalize to other settings, we will use industry average compensation costs for providers and staff to value time-driven costs as well as health system average compensation costs for the ROI analysis. Reimbursement data from the health system billing system will be collected for both the control and intervention period. We will collect clinic-level revenue as well as the Medicare allowed amounts for weight-prioritized visits. In addition, for the system-level ROI, we will obtain total system revenue for those patients that have a weight-prioritized visit in each calendar year. A practice perspective ROI will be calculated comparing the PATHWEIGH implementation cost to the difference in reimbursement for weight-prioritized visits during PATHWEIGH implementation and control periods. A system perspective ROI will compare the PATHWEIGH implementation cost to the difference in annualized revenue for patients with a weight-prioritized visit under the PATHWEIGH and control condition.

## Dissemination

PATHWEIGH was designed for dissemination [57]. The University of Colorado has trademarked PATHWEIGH so deployment through the large national network of Epic users is possible. This was not intended to generate revenue, but rather so data use agreements can be drafted that ensure data flow back to the study team so iterations can be made that improve its usefulness for all users. The e-learning module and practice facilitator training materials will be made available. Our team has experience with developing and disseminating implementation products [58]. Also, we will share results at scholarly meetings and in peer-reviewed journals. De-identified data sets used in analyses will be made available upon request.

## Trial status

This manuscript describes the study protocol (version 1, July 6, 2021) reviewed and funded by the National Institutes of Health (NIH) in September 2020. Enrollment to the study began in March 2021 and at the time of submission the research team is implementing PATHWEIGH in the first sequence of clinics. Recruitment and data collection will be completed in September 2025.

## Discussion

The goal of PATHWEIGH is to make weight management effective for patients and feasible and satisfying for medical providers and their teams in primary care. By utilizing the power of the electronic medical record to create efficiencies and drive evidence-based care, this goal may be achievable. This study will contribute to the field by testing if this approach accomplishes both effectiveness for patients’ health improvement and is adopted by typical clinical practices. It will add important findings about how the implementation strategies in combination contribute to both implementation and effectiveness outcomes. The use of a stepped wedge cluster randomized trial using covariate constrained randomization within the context of an effectiveness-implementation hybrid design is innovative and should advance methodological findings for the field. Although this study should provide findings that will be applicable to many settings and will be examined in a large number of diverse practices, this study will only be implemented in a single health system in one state. Therefore, the results may not translate to other circumstances and other settings, and contextual adaptation may be necessary. However, if successful, results may be disseminated through Epic, the most widely available electronic medical record system in the U.S. According to the Epic User Web, such a system is not yet available nor has been tested. Thus, this study could make a significant impact on weight and the resultant comorbidities experienced by Americans.

## Supplementary Information

**Additional file 1:** Covariate Constrained Randomization. **Fig. S1**. Standard of Care (Obesity brief HPI) initial and follow-up questionnaires.

## Data Availability

Not applicable.

## Abbreviations

BMI: Body mass index
CDC: Centers for Disease Control and Prevention
CME: Continuing medical education
EMR: Electronic medical record
FM: Family Medicine
fsQCA: Fuzzy-set Qualitative comparative analysis
GIM: General Internal Medicine
HPI: History of present illness
IBT: Intensive behavioral therapy
ICC: Intraclass correlation coefficient
MA: Medical assistant
NIH: National Institutes of Health
PRISM: Practical, Robust, Implementation and Sustainability Model
QCA: Qualitative comparative analysis
QI: Quality improvement
RE-AIM: Reach, Effectiveness, Adoption, Implementation, Maintenance
ROI: Return on investment
SD: Standard deviation
SOC: Standard of care
