# Supplementary material for: PATHWEIGH, pragmatic weight management in adult patients in primary care in Colorado, USA: study protocol for a stepped wedge cluster randomized trial
Source: Trials. 2022 Jan 10;23:26. doi: 10.1186/s13063-021-05954-7 (PMC8744030; doi:10.1186/s13063-021-05954-7)
Supplement: Supplementary file 1 — Additional file 1: Covariate Constrained Randomization. Fig. S1. Standard of Care (Obesity brief HPI) initial and follow-up questionnaires. [file 13063_2021_5954_MOESM1_ESM.docx]

**Additional File**

**Covariate Constrained Randomization**

The randomization was performed by the study biostatisticians (KS, LMD, EW). Location was categorized as sites in communities with <100,000 (rural) or $\geq$100,000 (urban). There were 5 sites with missing percentage Medicaid data, for which we imputed the median value of 3.2%. We simulated 100,000 randomization schemes from the total number of possible randomizations (~2x10^24^). Due to the small number of free-standing, non-hospital-based academic practices as well as rural practices, which are mutually exclusive, we generated randomizations that were stratified on these two variables. The remaining variables were then standardized and used to calculate a balance metric for each of the generated randomization schemes, where lower values indicate better balance of the covariates across the sequences. Each of these variables were assumed to have equal importance. For these selected randomizations, on average each pair of sites has 32% chance of appearing in the same sequence and appeared in different sequences 68% of the time, which is not far from the reference values of 33.3% (=1/3 waves) and 66.7%, respectively. Thus, we concluded that we did not overly constrain the randomization with our initial stratification approach and 20% balance metric cutoff and there is little loss in validity [1, 2].

**Fig. S1** Standard of Care (Obesity brief HPI) initial and follow-up questionnaires


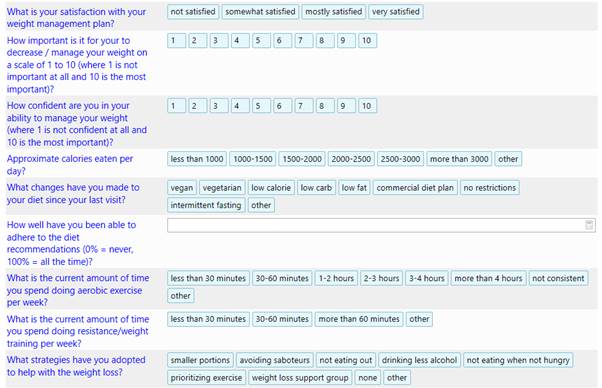


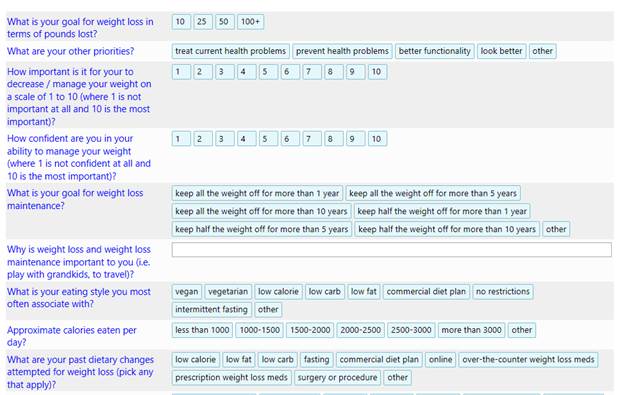


**References**

1. Bailey R, Rowley C. Valid randomization. Proceedings of the Royal Society of London A Mathematical and Physical Sciences. 1987;410(1838):105-24.

2. Moulton LH. Covariate-based constrained randomization of group-randomized trials. Clin Trials. 2004;1(3):297-305.
